# Rapid antigen testing in COVID-19 management for school-aged children: an observational study in Cheshire and Merseyside, UK

**DOI:** 10.1093/pubmed/fdac003

**Published:** 2022-02-04

**Authors:** David M Hughes, Sheila M Bird, Christopher P Cheyne, Matthew Ashton, Melisa C Campbell, Marta García-Fiñana, Iain Buchan

**Affiliations:** Department of Health Data Science, University of Liverpool, Liverpool, UK; MRC Biostatistics Unit, Cambridge, UK; Edinburgh University’s College of Medicine and Veterinary Medicine, Edinburgh, UK; Department of Health Data Science, University of Liverpool, Liverpool, UK; Department of Public Health, Liverpool City Council, Liverpool, UK; Department of Public Health, Liverpool City Council, Liverpool, UK; Department of Health Data Science, University of Liverpool, Liverpool, UK; Institute of Population Health Sciences, University of Liverpool, Liverpool, UK

**Keywords:** asymptomatic testing, COVID-19, lateral flow tests, rapid antigen tests, SARS-CoV-2, schools

## Abstract

**Background:**

Twice weekly lateral flow tests (LFTs) for secondary school children was UK Government policy from 8 March 2021. We evaluate use of LFTs (both supervised at test centres, and home test kits) in school-aged children in Cheshire and Merseyside.

**Methods:**

We report (i) number of LFT positives (ii) proportion of LFT positive with confirmatory reverse transcription polymerase chain reaction (PCR) test within 2 days, and (iii) agreement between LFT-positive and confirmatory PCR, and dependence of (i–iii) on COVID-19 prevalence.

**Findings:**

1 248 468 LFTs were taken by 211 255 12–18 years old, and 163 914 by 52 116 5–11 years old between 6 November 2020 and 31 July 2021. Five thousand three hundred and fourteen (2.5%) 12–18 years old and 1996 (3.8%) 5–11 years old returned LFT positives, with 3829 (72.1%) and 1535 (76.9%) confirmatory PCRs, and 3357 (87.7%) and 1383 (90.1%) confirmatory PCR-positives, respectively.

Monthly proportions of LFT positive with PCR negative varied between 4.7% and 35.3% in 12–18 years old (corresponding proportion of all tests positive: 9.7% and 0.3%).

Deprivation and non-White ethnicity were associated with reduced uptake of confirmatory PCR.

**Interpretation:**

Substantial inequalities in confirmatory testing need more attention to avoid further disadvantage through education loss. When prevalence is low additional measures, including confirmatory testing, are needed. Local Directors of Public Health taking more control over schools testing may be needed.

**Funding:**

DHSC, MRC, NIHR, EPSRC.

## Introduction

Lateral flow tests (LFTs) for SARS-CoV-2 antigen are widely used among other risk-mitigation measures in the UK’s response to the COVID-19 pandemic. LFTs may not be as sensitive as polymerase chain reaction (PCR) tests but provide a result within 30 minutes compared with 2–3 days for PCR, enabling prompt actions such as isolation. A population-wide study investigating the performance of Innova LFT versus PCR among people (mainly adults) not reporting symptoms of COVID-19 was carried out in Liverpool between 8 and 29 November 2020, reporting 40% LFT sensitivity compared with PCR overall, but with usefully higher sensitivity for identifying people with higher viral loads, more likely to be infectious.^1^^,^^2^ This was substantially lower than the performance of the device in the initial Public Health England (PHE) validation, but still potentially very useful—showing the need to evaluate end-to-end testing processes in real-world conditions.^3^^,^^4^ Reports of the performance of the Innova LFT in the Liverpool Community Testing pilot generated considerable debate, demonstrating the need to optimize utility such as time-to-isolate and proportion of the population reached, and not to consider diagnostic performance in isolation from the actions taken on test results.^5–13^

In the UK, for much of the pandemic, schools were closed to all except the children of key-workers. Part of the UK Government strategy to reopen schools has been regular LFT testing. Secondary school-aged children were expected to take school-monitored LFT tests twice a week from 8 to 19 March 2021; thereafter twice-weekly home-use of LFTs—a policy that has been hotly debated.^14–16^ Although welcomed by many seeking prompt action to minimize the indirect harms from COVID-19 restrictions, including loss of education, the lack of studies investigating the performance of Innova LFT in school-aged children was criticized.^15^^,^^16^ A PHE investigation found very low false positive rates (defined as the number of false positives divided by the total number of negatives, 9/1855 and 7/2130) in evaluations of Innova LFT in four secondary schools.^17^ Much of the debate about the utility of LFT has focused on false negatives and the risks of licencing behaviours that pose higher risks of SARS-CoV-2 transmission. However, increasing concern with the use of LFT in schools focussed on the proportion of discordant (LFT positive + later PCR negative) results, given that (at the time) classmates of individuals testing positive needed to quarantine (if no alternative arrangement such as daily test-to-release was in place).^5^ This can substantially impair children’s education. The Department of Health and Social Care reported proportions of positive LFT paired with negative PCRs within 2 days among secondary school children of 62% and 55% for the weeks 4–10 March and 11–17 March, the first 2 weeks of the twice weekly testing in schools policy.^18^^,^^19^ During these weeks 1331 and 1766 positive LFTs were observed giving 480 and 466 positives per million LFT tests, respectively, with 48% and 43% of positive LFTs obtaining a confirmatory PCR within 2 days. However, the proportion of positive LFTs that are linked to a negative PCR depend largely on the prevalence at the time of testing.^8^ Between 8 March and 29 March 2021, Government policy was that positive LFTs performed within secondary schools (as opposed to at home) would not require confirmatory PCR testing, and that a subsequent negative PCR would not remove isolation/quarantine requirements for individuals/close contacts.^14^

A national pilot of open-access LFT testing was introduced in the City of Liverpool (0.5 m population) on 6 November 2020, to provide rapid testing to people without common symptoms of COVID-19. This testing expanded to the wider Liverpool City Region (1.5 m population), followed by all of Cheshire and Merseyside (2.6 m population) in December 2020. This paper describes details of LFT testing in school-aged children in Cheshire and Merseyside, characterizes the change in uptake and positivity rate over time and investigates how many individuals with positive LFT results have a confirmatory PCR within 2 days, and the results of PCR within 2 days. We provide the first large scale, population/system-wide report of LFT testing in school-aged children in a period that covers both Alpha and Delta variants and provides evidence to assess the utility of LFT testing in schools. Routine asymptomatic testing of 5–11 was not part of the schools testing programme and has never been government policy. Nevertheless, since reasonable numbers of 5–11 years old have been tested we also report on this group to give a comprehensive summary of LFT testing in children.

## Methods

### Study design and participants

The data for this study come from the Combined Intelligence for Population Health Action (CIPHA; www.cipha.nhs.uk) data resource. Data used in this study included all Pillar 2 LFT and PCR tests conducted in Cheshire and Merseyside between 6 November 2020 and 31 July 2021. LFT tests were conducted either at asymptomatic test centres, or through test kits delivered to individual households. Tests performed at test centres were performed by the individual but interpreted and recorded by the test centre staff whereas home tests were performed, interpreted and recorded by the individual. PCR tests, whether at test centres or home tests, were sent to Lighthouse Laboratories for PCR testing using their standard ThermoFisher TaqPath™ RT-PCR SARS-CoV-2 assay. Demographic data were available on the tested individuals, including age, sex, ethnicity (coded as ‘Black’, ‘White’, ‘Asian’, ‘Mixed or multiple ethnic groups’, ‘Another ethnic group’ or ‘Prefer not to say’) and lower layer super output area.

We consider any test taken by an individual aged 5–11 as being a test on a primary school-aged child and any test taken by an individual aged 12–18 as being a test on a secondary school-aged child. This is not a precise definition as it will inevitably include some tests on individuals who are not at school (e.g. 18-year-old university students) or some children who are classified incorrectly (e.g. 11 years old who have just started secondary school). However, we do not have specific data on testing in schools and so can only report on tests by the reported age. We use the designation of primary and secondary school-aged children for the purposes of illustration.

### Statistical analysis

We report the LFT positivity and void proportions from all LFTs performed daily since 6 November 2020, and the proportion of all positive LFTs that have a confirmatory PCR within 2 days of the positive result. For individuals with a positive LFT and a confirmatory PCR we report the proportion of PCR tests that were positive. Ninety-five percent confidence intervals for proportions were calculated using the Clopper–Pearson exact method. Void PCR results were considered discordant in this analysis, to be conservative in reporting the agreement between positive LFT and confirmatory PCR.^20^ Given the very low numbers of void PCR results, this is unlikely to alter the reported values substantially.

We define discordant confirmatory positives as LFT +ve then PCR −ve within 2 days—colloquially/loosely ‘false positives’. However, we acknowledge that PCR is not a gold standard: swabbing is not completely reliable for picking up viral genetic material from an infected person, and laboratory/logistic processes are not fully reliable.

Multifactorial logistic regression was applied to assess factors influencing (i) uptake of confirmatory PCR tests following positive LFT and (ii) agreement between positive LFTs and confirmatory PCR. Each model included age, sex, ethnicity, IMD deprivation quintile, whether the LFT was a self-reported home test and the number of LFTs undertaken in the 2 weeks prior to a positive LFT as possible explanatory covariates. For model (ii) we coded ethnicity as White/non-White/prefer not to say due to small numbers in some of the groups.

Where multiple positive LFT tests were reported for a single individual, we selected the first positive test that also had a confirmatory PCR (so as to identify as many confirmatory PCRs as possible), or the first positive test if no confirmatory PCRs were observed. Analysis was performed separately for primary and secondary school-aged children. Thirteen 12–18 years old, and one 5–11 years old had unknown sex and were omitted from the regression models.

## Results

Between 6 November 2020 and 31 July 2021, 163 941 and 1 248 468 LFTs were taken by 5–11- and 12–18 years old, respectively in Cheshire and Merseyside. This identified 2065 (1.3%) and 5564 (0.4%) positive tests representing 1996 and 5314 individuals aged 5–11 and 12–18, respectively.

LFT use increased substantially amongst school-aged children at the beginning of March, coinciding with the return to school for most children in the UK (Supplementary Fig. S1). Interestingly, although LFT testing was only government policy in secondary schools, a noticeable increase in LFT tests in 5–11 years old was also observed. After the initial period of schools-based testing (2 weeks) uptake of LFT dropped substantially, as lateral flow testing in secondary school-aged children moved mostly to home testing. However, use of LFTs in school-aged children was still noticeably higher than before the UK government advised twice-weekly testing. Uptake of PCR testing is shown in Supplementary Fig. S2.

Government guidance (from 29 March onwards) suggests 2 days as the time window for confirmatory PCR following a positive LFT.^14^ Not all individuals with a positive LFT undertook a confirmatory PCR, with 76.9% of 5–11 years old and 72.1% of 12–18 years old taking a PCR test within 2 days of a positive LFT (Supplementary Table S1). Altering the observed time window for a confirmatory PCR to allow up to 5 days for a confirmatory PCR gave similar results (see supplementary material). Supplementary Fig. S3 details the number of positive LFTs obtained each day, as well as how many of the positive LFTs received a confirmatory PCR and the subsequent result.

**Fig. 1:**
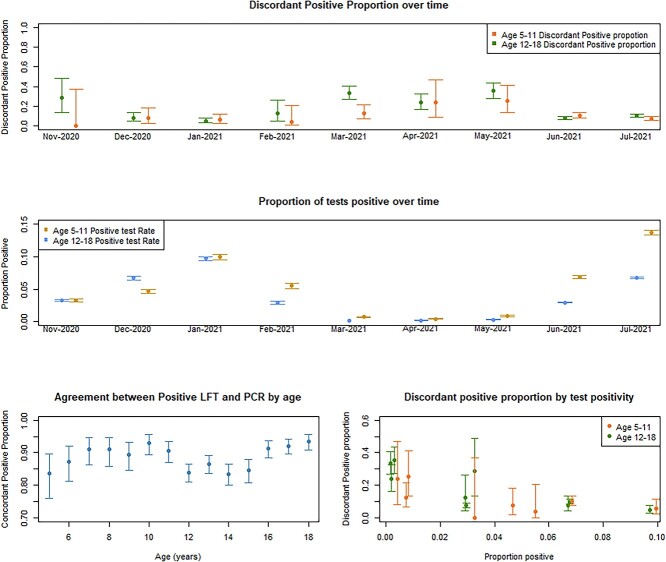
Proportion of disagreement (with 95% confidence intervals) between positive LFT and confirmatory PCR, over time (top panel), proportion of all tests positive over time (middle panel), concordant positive proportion by age (bottom left) and the relationship between discordant positive proportion and proportion of all tests positive (bottom right). Confirmatory PCR was determined as a PCR within a 2-day window of the positive LFT. The proportion of tests positive was calculated as all positives (either PCR or LFT) divided by all tests taken within the month for each age group. We have assumed, conservatively, that all void PCR results are discordant positives.

Approximately 87.7% (95% CI: 86.6%, 88.7%) of confirmatory PCR’s in 12–18 years old and 90.1% (95% CI: 88.5%, 91.5%) in 5–11 years old were positive (Table S1). Excluding void PCR results did not substantially change these results. There was some variation with age in the proportion of positive LFTs that are concordant with PCR, with lower agreement for 12–15 years old compared to 16–18 years old (Fig. 1, bottom left panel). The relationship between age and agreement between positive LFT and confirmatory PCR is less clear in 5–11 years old.

The overall disagreement between positive LFT and confirmatory PCR was around 9.9% in 5–11 years old (8.5%, 11.5%) compared with 12.3% (11.3%, 13.4%) in 12–18 years old. Beneath these summaries there was substantial variability across the study period with higher proportions of disagreement when the prevalence of COVID-19 was lower (Fig. 1). Table 1 describes the testing results per month between November 2020 and July 2021. From late March onwards most positive LFTs were detected using home test kits. The proportion of LFT positives that were linked to a negative PCR was higher when the proportion of positive tests was lower implying a lower prevalence (Fig. 1 bottom right). Supplementary Table S2 shows equivalent data for 5–11 years old.

**Table 1:** Description of testing results in 12–18 years old by month between November 2020 and July 2021

Month	Nov-20	Dec-20	Jan-21	Feb-21	Mar-21^*^	Apr-21	May-21	Jun-21	Jul-21
Positive test percentage	3.26%	6.70%	9.71%	2.91%	0.15%	0.20%	0.31%	2.96%	6.77%
Total registered LFTs	29 560	14 163	27 179	23 723	510 977	200 790	173 095	154 646	114 335
Total individuals with LFT	19 977	11 833	16 775	12 210	158 094	67 008	47 556	51 063	46 288
Total individuals with positive LFT	126 (0.6%)	213 (1.8%)	454 (2.7%)	111 (0.9%)	389 (0.3%)	226 (0.3%)	233 (0.5%)	1435 (2.8%)	2127 (4.6%)
Total confirmatory PCR	28 (22%)	158 (74%)	291 (64%)	41 (37%)	203 (52%)	107 (47%)	144 (62%)	1165 (81%)	1692 (80%)
Confirmatory PCR positive	20 (71%)	144 (91%)	275 (95%)	35 (85%)	131 (65%)	83 (78%)	95 (66%)	1070 (92%)	1504 (89%)
Confirmatory PCR Negative	8 (29%)	12 (8%)	14 (5%)	5 (12%)	69 (34%)	23 (22%)	49 (34%)	89 (8%)	165 (10%)
Confirmatory PCR void	0 (0%)	2 (1%)	2 (1%)	1 (2%)	3 (1%)	1 (1%)	0 (0%)	6 (1%)	23 (1%)
Total LFT positive home tests (% of all LFT positives)	0 (0%)	0 (0%)	6 (1%)	12 (11%)	245 (63%)	218 (96%)	222 (95%)	1353 (94%)	1969 (93%)
Confirmed LFT positive home tests (% of all positive home tests)	0 (0%)	0 (0%)	2 (33%)	5 (42%)	135 (55%)	100 (46%)	137 (62%)	1117 (83%)	1592 (81%)
LFT positive home test with positive PCR	0 (0%)	0 (0%)	2 (100%)	4 (80%)	90 (67%)	78 (78%)	90 (66%)	1027 (92%)	1443 (91%)
Total LFT positives at test centres (% of all LFT positives)	126 (100%)	213 (100%)	448 (99%)	99 (89%)	144 (37%)	8 (4%)	11 (5%)	82 (6%)	158 (7%)
Confirmed LFT positives from test centres (% of all positive test centre tests)	28 (22%)	158 (74%)	289 (65%)	36 (36%)	68 (47%)	7 (88%)	7 (64%)	48 (59%)	100 (63%)
LFT positives from test centre with positive PCR	20 (71%)	144 (91%)	273 (94%)	31 (86%)	41 (60%)	5 (71%)	5 (71%)	43 (90%)	61 (61%)

Positive test percentage is defined as the percentage of all tests (PCR or LFT) that are positive.

^*^Government guidance changes to twice weekly LFT testing for secondary school children.

Self-reported positive LFTs were more likely to obtain a confirmatory PCR than supervised tests (odds ratio (OR) 3.48 (2.68, 4.52) in 5–11 years old and 2.16 (1.86, 2.50) in 12–18 years old). Most non-White children were less likely to obtain confirmatory PCRs than White children, with most associations statistically significant in 12–18 years old, although only Black children having a statistically significant association amongst 5–11 years old (Table 2). Children in the most deprived quintile were less likely to obtain a confirmatory PCR following a positive LFT than children living in the least deprived quintile. Forty-five percent of 5–11 years old and 38.3% of 12–18 years old with positive LFT lived in the most deprived areas of Cheshire and Merseyside. Increasing age decreased (approximately linearly) the likelihood of having a confirmatory PCR (Supplementary Fig. S4). Similar ORs were obtained for both 5–11- and 12–18 years old but statistical significance was usually only obtained in 12–18 years old, likely due to the increased sample size (Table 2). An individual’s sex, and the number of LFTs taken in the last 14 days were not significant predictors of whether an individual would get a confirmatory PCR following a positive LFT.

**Table 2:** Odds ratios from multiple logistic regression models showing the association with uptake of confirmatory PCR test after a positive LFT

Explanatory variable			Primary schools (aged 5–11)			Secondary school (aged 12–18)			
		Summary	Odds ratio	95% CI	*P*-value	Summary	Odds ratio	95% CI	*P*-value
Intercept			2.33	(1.53, 3.61)			1.89	(1.52, 2.34)	
Age		9 (7, 10)	0.98	(0.92, 1.03)	0.422	15 (13, 17)	0.96	(0.93, 0.99)	0.013
Sex	Reference = Female	1014 (50.8%)				2755 (52.0%)			
	Male	981 (49.2%)	0.87	(0.70, 1.09)	0.221	2546 (48%)	1.09	(0.96, 1.23)	0.173
Ethnicity	Reference = White	1692 (84.8%)				4544 (85.7%)			
	Asian	52 (2.6%)	0.84	(0.44, 1.69)	0.610	116 (2.2%)	0.42	(0.29, 0.61)	<0.001
	Black	20 (1.0%)	0.39	(0.15, 0.99)	0.043	68 (1.3%)	0.35	(0.21, 0.58)	<0.001
	Mixed or multiple ethnic groups	83 (4.2%)	1.79	(0.94, 3.79)	0.096	153 (2.9%)	0.88	(0.61, 1.28)	0.489
	Another ethnic group	8 (0.4%)	0.77	(0.16, 5.67)	0.765	19 (0.3%)	0.27	(0.10, 0.67)	0.005
	Prefer not to say	140 (7.0%)	1.00	(0.67, 1.51)	0.997	401 (7.6%)	0.70	(0.56, 0.88)	0.002
IMD deprivation quintile	Reference = 5 (least deprived)	258 (12.9%)				868 (16.4%)			
	1 (most deprived)	898 (45.0%)	0.45	(0.30, 0.65)	<0.001	2033 (38.3%)	0.67	(0.55, 0.81)	<0.001
	2	294 (14.7%)	0.84	(0.52, 1.32)	0.447	767 (14.5%)	0.85	(0.67, 1.06)	0.154
	3	253 (12.7%)	0.95	(0.58, 1.54)	0.827	739 (13.9%)	1.01	(0.80, 1.28)	0.946
	4	292 (14.6%)	0.84	(0.52, 1.34)	0.473	894 (16.9%)	1.06	(0.84, 1.33)	0.641
Total LFTs in previous 14 days		0 (0, 0)	0.91	(0.79, 1.06)	0.226	0 (0, 1)	0.96	(0.91, 1.01)	0.114
Was the LFT a self-reported home test?	Reference = No	423 (21.2%)				1288 (24.3%)			
	Yes	1573 (78.8%)	3.48	(2.68, 4.52)	<0.001	4013 (75.7%)	2.16	(1.86, 2.50)	<0.001

For categorical variables we report the number and percentage, whereas for continuous variables we report the median and interquartile range. Age has been centred on 15 in secondary school children and eight in primary school-aged children.

Positive LFTs on older children were more likely to be confirmed with a positive PCR, with an increase in odds of 17% (OR: 1.17; 1.12, 1.23) per year of age in 12–18 years old and 10% (OR 1.10, 1.01, 1.20) per year in 5–11 years old. Individuals aged 12–18 with positive LFTs who had more LFTs in the 14 days prior to a positive LFT were less likely to have a positive PCR. Supplementary Fig. S7 shows that there was some non-linearity in this relationship, although the smaller numbers of individuals with more than two tests in the 14 days prior to a positive LFT make it challenging to assess this non-linearity (Supplementary Tables S5 and S6). Self-reported positive LFTs were more likely to agree with the PCR compared to testing site supervised tests, although statistical significance was only obtained in 12–18 years old (Table 3). We suspect this is due to confounding with time, as most of the self-reported tests occurred in June and July 2021 when COVID-19 prevalence was high and thus discordant positives less likely (Table 2). Deprivation was not a significant predictor of whether the confirmatory PCR would agree with the original positive LFT in primary school-aged children. There was not a strong signal in secondary school-aged children but a suggestion of a small association when comparing the most deprived with the least deprived areas.

**Table 3:** Odds ratios for a multiple logistic regression showing the association with agreement between positive LFT and confirmatory PCR

Explanatory variable		Primary schools (aged 5–11)				Secondary school (aged 12–18)			
		Summary	Odds ratio	95% CI	*P*-value	Summary	Odds ratio	95% CI	*P*-value
Intercept			10.22	(5.17–21.45)			6.66	(4.72, 9.52)	
Age		9 (7, 10)	1.10	(1.01, 1.20)	0.024	15 (13, 17)	1.17	(1.11, 1.23)	<0.001
Sex	Reference = Female	788 (51.4%)				1965 (51.4%)			
	Male	746 (48.6%)	1.41	(1.00, 1.99)	0.050	1858 (48.6%)	1.04	(0.86, 1.27)	0.676
Ethnicity	White	1324 (86.1%)				3382 (88.5%)			
	Non-White	128 (8.3%)	1.52	(0.79, 3.29)	0.246	215 (5.6%)	1.01	(0.67, 1.58)	0.968
	Prefer not to say	82 (5.34%)	0.78	(0.36, 1.92)	0.567	226 (5.9%)	0.82	(0.56, 1.21)	0.304
IMD deprivation quintile	Reference =5 (least deprived)	220 (14.3%)				673 (17.6%)			
	1 (most deprived)	622 (40.5%)	1.01	(0.58, 1.68)	0.985	1341 (35.1%)	0.81	(0.59, 1.09)	0.163
	2	239 (15.6%)	0.89	(0.48, 1.63)	0.701	552 (14.4%)	0.67	(0.47, 0.95)	0.023
	3	211 (13.8%)	0.92	(0.49, 1.74)	0.796	563 (14.7%)	0.88	(0.61, 1.26)	0.470
	4	242 (15.8%)	1.08	(0.57, 2.04)	0.813	694 (18.2%)	1.05	(0.74, 1.49)	0.798
Total LFTs in previous 14 days		0 (0, 0)	1.05	(0.83, 1.41)	0.703	0 (0, 1)	0.93	(0.86, 1.00)	0.035
Was the LFT a self-reported home test?	Reference = No	237 (15.4%)				740 (19.4%)			
	Yes	1297 (84.6%)	0.68	(0.38, 1.16)	0.180	3083 (80.6%)	1.57	(1.22, 2.02)	<0.001

For categorical variables we report the number and percentage, whereas for continuous variables we report the median and interquartile range. Age has been centred on 15 in secondary school children and eight in primary school-aged children.

Differences in the cycle threshold (Ct) values of confirmatory PCRs by age were small, with younger children generally having higher Ct (hence lower viral load, Supplementary Fig. Fig. S5). More substantial differences were evident over time suggesting individuals testing positive with suspected Delta variant had higher viral loads than suspected Alpha (Supplementary Fig. Fig. S6).

## Discussion

### Main findings of this study

This study reports nine months of LFT testing in school-aged children for a whole population over both Alpha and Delta variant surges of the COVID-19 pandemic and provides important implementation evidence for measuring and managing the utility of LFT testing in schools. LFT testing identified over 5000 12–18 years old and almost 2000 5–11 years old with positive results. It is likely that most of these children were identified earlier than if they had waited to display symptoms and seek a PCR test. The resulting isolation periods are likely to have reduced the onwards transmission of COVID-19. However, we note that isolating, not just of the individual, but also (previously) of their classmates will inevitably have some effect their education. For this reason, confirmatory testing is crucial, and it is important that inequalities in the uptake of confirmatory testing are addressed.

We show that although >80% positive LFTs were confirmed by a positive PCR, there were times when the observed proportion of positive LFTs followed by a negative PCR within 2 days approached 40%. These discordant results are often termed ‘false positives’, however, we note that typical swabbing is not 100% reliable at picking up viral genetic material from an infected person, and that laboratory processing/logistics are not without error.

### What is already known on this topic

Most concerns about the use of LFTs have focussed on false negatives and the consequences of cases missed.^15^ However, in schools testing, and at times of low prevalence of SARS-CoV-2, false positives attracted more attention as they can lead to unnecessary loss of schooling for cases and large numbers of their class/group-based contacts.^18^ Our study shows that the proportion of LFT positives followed in 2 days by a negative PCR changed substantially with test positivity rates during the study period—positivity reflecting prevalence of SARS-CoV-2, alongside general population case rates. This is consistent with previous findings reporting proportions of positive LFT linked to a negative PCR of <10% when prevalence was high^2^ and around 35% when the prevalence of SARS-CoV-2 was low (although the DHSC proportions for the first 2 weeks of testing in schools were even higher—more than half—than we observed in our cohort at 50–60%).^19^

The most deprived areas of Cheshire and Merseyside were the least likely to have a confirmatory PCR test following a positive LFT, but most deprived have 2.5 times as many LFT positives as the least deprived quintile. This extends previous findings of lower testing uptake in disadvantaged areas of Cheshire and Merseyside.^21^ Similarly, non-White children appeared less likely to take up confirmatory PCRs. In both cases, policy makers should consider ways of encouraging greater uptake of confirmatory PCRs amongst these groups.

### What this study adds

Policy makers should give careful thought to adapting testing regimens according to background prevalence and risk contexts such as the proportion of general population vaccinated.^22^ In our cohort, we observed 5314 positive individuals using almost 1.25 million tests (~4.25 positives per 1000 tests) in secondary school-aged children. Vaccination was introduced and reached substantial adult coverage over our study period, although children were not vaccinated. Given that in periods of lower prevalence a larger proportion of the LFT positives will be PCR-discordant, a more targeted approach to testing may yield greater value, especially as the consequences of transmission are quite different where adult populations are largely vaccinated. We recommend policy makers support rapid evidence generation on routinely collected data such as CIPHA, and that testing cost data are included to enable rapid economic evaluations.

Some individuals (around 3.6% in 12–18 years old) appear to take a second LFT instead of organizing a confirmatory PCR, and that most of these second LFTs did not agree with the original positive. Further details of this are provided in the supplementary material.

In our observation period, most LFT testing employed the Innova SARS-CoV-2 rapid antigen lateral flow device. Towards the end of the period, increasingly from June 2021, schools-based testing moved to the Orient Gene device. Trials using the Orient Gene device have been reported.^17^

### Limitations of this study

Our study has several limitations. Only around 77% of 5–11 years old and 72% of 12–18 years old had a confirmatory PCR after a positive LFT. For a small number of students this was probably because Government policy since end of January 2021 to 29 March 2021 was not to confirm positive LFTs with a PCR.^15^ Nevertheless, this increases the level of uncertainty (and possibly bias) in the estimates of the proportion of false positive here reported since we do not know whether a PCR would have confirmed the remaining LFT positives or not.

Another potential source of bias is the use of fake analyte by children seeking a ‘false positive’ test result to engineer a day off school. A recent study reported 10 out of 14 soft drinks could be used to produce a positive Innova LFT result.^23^ This could increase the number of positive LFTs that do not have a positive PCR.

LFT testing carries risks of unnecessary isolation, quarantine and education loss. Mitigating these risks with confirmatory testing is not yet fully realized. Daily LFT testing has recently been reported as an alternative to quarantine for class/group-based contacts of cases.^24^ Data on school attendance following a positive LFT are not routinely linked or analysed, yet may help inform local policies. We cannot assess the extent to which LFT testing in schools prevents transmission. Considering the findings of our study and the further scope to use routinely collected data to adapt COVID-19 responses around schools, we recommend giving Directors of Public Health more control to vary testing policies according to local contexts.

## Authors’ contributions

DMH, MGF and IB designed the study. DMH managed the data and conducted the initial analysis, under supervision of MGF and IB and in discussion with SMB. CPC checked the analysis. MGF, DMH and IB drafted the initial manuscript. All authors contributed to the writing of the manuscript. The corresponding author attests that all listed authors meet authorship criteria and that no others meeting the criteria have been omitted.

## Data availability

Pseudonymised data are accessible via CIPHA. Requests can be made to the Data Asset and Access Group for extracts of the larger scale data which cannot be released openly due to information governance requirements. All R code is accessible from the corresponding author.

## Transparency

The lead author (the manuscript’s guarantor) affirms that the manuscript is an honest, accurate and transparent account of the study being reported; that no important aspects of the study have been omitted; and that any discrepancies from the study as planned (and, if relevant, registered) have been explained.

## Supplementary Material

STROBE_checklist_v4_combined_JPH_SchoolsTesting_fdac003

## Notes

Denotes shared last authorship.

## Notes

**David M. Hughes**, Lecturer in Health Data Science.

**Sheila M. Bird**, Honorary Professor of Biostatistics.

**Christopher P Cheyne**, Postdoctoral Research Associate.

**Matthew Ashton**, Director of Public Health.

**Melisa C. Campbell**, Consultant in Public Health (Children and Young People).

**Marta García-Fiñana**, Professor of Health Data Science.

**Iain Buchan**, Professor of Public Health and Clinical Informatics.
